# Sedentary behavior, body image, physical activity enjoyment, and life satisfaction: a three-wave longitudinal study among college students

**DOI:** 10.3389/fpubh.2026.1894305

**Published:** 2026-10-21

**Authors:** Liyan Wu, Guoqiang Sang, Bodi Zhang, Yonglian Li

**Affiliations:** 1 Faculty of Sports and Health, Wenzhou University, Wenzhou, China; 2 College of General Education, Zhejiang College of Security Technology, Wenzhou, China

**Keywords:** body image, college students, life satisfaction, longitudinal study, physical activity enjoyment, sedentary behavior

## Abstract

**Background:**

Excessive sedentary behavior is a modifiable public health concern in university populations, and life satisfaction is an important indicator of psychological wellbeing and a relevant target for campus health promotion. This study examined the longitudinal association between sedentary behavior and life satisfaction and tested whether body image and physical activity enjoyment operated as parallel psychological mediators.

**Methods:**

A three-wave longitudinal survey was conducted among college students in Zhejiang Province, China, at six-month intervals. The analytic sample comprised 426 participants with complete matched data across all three waves. The measurement model represented body image (BI) and physical activity enjoyment (PACES) with four item parcels each and life satisfaction (SWLS) with its five individual items. Longitudinal mediation controlled both T2 and T1 SWLS, and a fully reciprocal manifest-score cross-lagged panel model (CLPM) together with a full-forward lag-2 robustness model was estimated.

**Results:**

T1 sedentary behavior negatively predicted T2 body image (β = **–**0.227, *p* < 0.001) and physical activity enjoyment (β = **–**0.248, *p* < 0.001). After adjustment for T2 and T1 life satisfaction, T2 body image (β = 0.151, *p* < 0.001) and physical activity enjoyment (β = 0.220, *p* < 0.001) independently predicted T3 life satisfaction. Bias-corrected bootstrap analyses showed significant indirect effects through body image [*b* = **–**0.150, 95% CI (**–**0.247, **–**0.079)] and physical activity enjoyment [*b* = −0.237, 95% CI (−0.368, −0.141)]; their difference was not significant. The fully reciprocal manifest-score CLPM showed acceptable overall fit (χ^2^ = 45.397, df = 16, CFI = 0.989, TLI = 0.954, RMSEA = 0.066, SRMR = 0.020).

**Conclusions:**

Sedentary behavior was longitudinally associated with lower life satisfaction among college students through both body-related self-evaluation and exercise-related affective experience. From a public health and health-promotion perspective, the findings identify prolonged sitting as a potentially modifiable behavioral target and support integrated campus strategies that combine sedentary-time reduction, positive body-image promotion, and enjoyable physical activity opportunities to support student wellbeing.

## 1 Introduction

Sedentary behavior has become a prominent public health concern in university settings because contemporary student life involves prolonged academic sitting, screen-based learning, smartphone use, and sedentary leisure. A systematic review and meta-analysis of 125 studies estimated that university students accumulate approximately 7.29 h/day of self-reported sedentary time, with device-based estimates approaching 9.82 h/day (1). Importantly, sedentary behavior is not simply the absence of physical activity: students may meet exercise recommendations while still spending a large proportion of their waking day sitting (2, 3). This distinction makes sedentary behavior a potentially modifiable health-promotion target in its own right.

Life satisfaction, the cognitive component of subjective wellbeing, reflects an individual's global evaluation of life quality and circumstances (4, 5). During the university years, life satisfaction is closely related to academic adjustment, emotional functioning, social relationships, and longer-term psychological health. From a public health perspective, maintaining life satisfaction in young adults is therefore relevant not only to individual wellbeing but also to health education and prevention strategies within higher-education environments. Multinational evidence further indicates that sedentary behavior and physical activity are independently and jointly associated with life satisfaction, happiness, and perceived health among university students (6, 33)

Among the behavioral factors that may shape life satisfaction, excessive sedentary time warrants particular attention. Prolonged sitting may reduce opportunities for movement, social engagement, embodied competence, and positive affective experiences, thereby influencing how students evaluate their lives. Existing evidence suggests an inverse association between sedentary behavior and wellbeing (6), but the psychological processes underlying this relationship remain insufficiently understood. Clarifying these mechanisms is important for health promotion because interventions are more likely to be effective when they address not only a risk behavior itself but also the psychological pathways through which that behavior is associated with wellbeing.

Body image is one plausible self-evaluative pathway. Body image encompasses perceptions, evaluations, and emotional attitudes toward one's appearance, shape, weight, and physical functioning (7, 8). University students are particularly exposed to appearance-related social comparison, identity development, and gendered body ideals. Prolonged sedentary behavior may be associated with less favorable body perceptions by weakening perceived physical competence and embodied vitality or increasing concern about shape and weight. In turn, less positive body image may reduce self-acceptance and global life evaluation. Prior studies have linked body image to life satisfaction and broader psychological adjustment (9), and body image has been identified as a mediator between physical exercise and subjective wellbeing in college students (10).

Physical activity enjoyment represents a second, affective-motivational pathway. Enjoyment refers to the pleasure, interest, satisfaction, and intrinsic reward experienced during physical activity (11). Self-determination theory proposes that intrinsically motivated behavior is more likely to be maintained when individuals experience autonomy, competence, and positive affect, all of which are closely connected to wellbeing (12). Consistent with this framework, autonomous motivation is associated with physical activity participation and maintenance (13), and enjoyment predicts exercise habit, intention to continue exercising, and exercise frequency (14). A highly sedentary lifestyle may make movement feel less familiar, less competent, or more effortful, thereby reducing enjoyment and weakening a potentially important source of positive affect and life satisfaction.

Although body image and physical activity enjoyment may be correlated, they represent conceptually different mechanisms and are therefore modeled as parallel rather than sequential mediators. Body image primarily reflects body-related self-perception and evaluation, whereas physical activity enjoyment reflects affective and motivational experience during movement. Sedentary behavior may consequently be associated with life satisfaction through both a self-evaluative pathway and an affective-motivational pathway. Testing these mediators simultaneously provides a more differentiated account of the psychological processes relevant to campus health promotion without assuming a fixed causal order between body perception and exercise-related affect.

Several gaps in the literature justify a longitudinal test of this model. First, much of the evidence linking sedentary behavior, body image, physical activity, and wellbeing is cross-sectional, which limits temporal interpretation and can bias mediation estimates when the underlying processes unfold over time (15). Second, prior research has emphasized physical activity as a protective behavior more often than sedentary behavior as an independent behavioral exposure. Third, few longitudinal studies have examined body image and physical activity enjoyment simultaneously as conceptually distinct mediating pathways. Addressing these gaps requires a repeated-measures design that separates the timing of the predictor, mediators, and outcome and reports the longitudinal measurement and structural specifications transparently.

Based on the theoretical and empirical evidence above, the following hypotheses were proposed:

**H1**. Higher sedentary behavior will predict lower subsequent life satisfaction among college students after accounting for baseline life satisfaction and relevant covariates.

**H2**. Body image will mediate the longitudinal association between sedentary behavior and life satisfaction, such that higher sedentary behavior will predict less positive body image, which in turn will predict lower subsequent life satisfaction.

**H3**. Physical activity enjoyment will mediate the longitudinal association between sedentary behavior and life satisfaction, such that higher sedentary behavior will predict lower physical activity enjoyment, which in turn will predict lower subsequent life satisfaction.

**H4**. Body image and physical activity enjoyment will operate as parallel mediators between sedentary behavior and life satisfaction, representing distinct self-evaluative and affective-motivational pathways.

Accordingly, the objective of this three-wave longitudinal study was to determine whether sedentary behavior prospectively predicts life satisfaction among college students and whether body image and physical activity enjoyment explain this association as parallel mediators. The study additionally estimated a fully reciprocal manifest-score CLPM to characterize prospective between-person ordering and used a full-forward model with T1 → T3 lag-2 paths as a robustness analysis.

## 2 Materials and methods

This study adopted a three-wave longitudinal design to examine the association between sedentary behavior and life satisfaction among college students and to test the parallel mediating roles of body image and physical activity enjoyment. Participants were recruited from universities in Zhejiang Province, China, and included full-time undergraduate and postgraduate students.

Participants were eligible if they were aged 18 years or older, were currently enrolled at a university, were able to complete Chinese-language questionnaires independently, provided informed consent, and agreed to participate in all three waves of the survey. Participants were excluded if they had severe cognitive impairment or psychiatric conditions that prevented independent completion of the questionnaire, were participating in intervention programs that might directly affect sedentary behavior, physical activity, body image, or life satisfaction, or provided invalid questionnaire responses.

A total of 526 eligible students completed the baseline assessment. At the six-month follow-up, 476 students remained in the study, and 426 completed the final assessment twelve months after baseline. Thus, the final analytic sample included 426 students, with an overall retention rate of 81.0%. Among them, 188 were male, and 238 were female. The mean age was 20.54 years (SD = 1.57), and the mean BMI was 21.75 kg/m^2^ (SD = 2.73). The sample included 327 undergraduate students, 59 master's students, and 40 doctoral students. The final sample size was adequate for the planned longitudinal structural equation modeling and mediation analyses.

### 2.1 Procedure

Participants were recruited through university student affairs offices, academic departments, physical education divisions, campus bulletin boards, class-based announcements, and online student communities such as WeChat and QQ groups. Before data collection, all participants received a detailed explanation of the study purpose, survey procedures, voluntary nature of participation, confidentiality safeguards, potential risks, and their right to withdraw from the study at any time without penalty. Written or electronic informed consent was obtained from all participants before they completed the baseline questionnaire.

Data were collected at three time points, with an interval of approximately six months between adjacent waves. At Time 1 (T1), participants completed questionnaires assessing sedentary behavior, body image, physical activity enjoyment, life satisfaction, physical activity level, and demographic characteristics. At Time 2 (T2), approximately six months after baseline, participants again completed measures of sedentary behavior, body image, physical activity enjoyment, and life satisfaction. At Time 3 (T3), approximately twelve months after baseline, the same core study variables were assessed again. This repeated-measures design allowed the study to examine temporal associations among sedentary behavior, body image, physical activity enjoyment, and life satisfaction, and provided the basis for longitudinal measurement invariance testing, cross-lagged panel analysis, and longitudinal mediation analysis.

The primary mediation model examined whether T1 sedentary behavior predicted T3 life satisfaction through two parallel psychological pathways: T2 body image and T2 physical activity enjoyment. In this model, sedentary behavior at T1 was specified as the independent variable, body image and physical activity enjoyment at T2 were specified as parallel mediators, and life satisfaction at T3 was specified as the outcome variable. Baseline levels of body image, physical activity enjoyment, and life satisfaction were controlled to reduce the influence of prior individual differences.

To match responses across the three waves while preserving anonymity, each participant was assigned a unique research code. The code was used only for longitudinal data matching and did not contain personally identifiable information. All questionnaire data were stored in password-protected files accessible only to the research team. In all reports and publications, only aggregated statistical results were presented, and no information that could identify individual participants was disclosed.

To reduce fatigue and careless responding, the questionnaire was designed to take approximately 15–25 min to complete at each wave. Participants were informed that they could discontinue the survey at any time. At the end of each questionnaire, brief health education information was provided to encourage students to reduce prolonged sitting and increase light-intensity movement in daily life. For participants who experienced discomfort when responding to questions about body image or lifestyle habits, information about university's psychological counseling services was provided.

The study protocol was reviewed and approved by the Ethics Committee of Wenzhou University before data collection. All procedures were conducted in accordance with the Declaration of Helsinki. Approval No.WZU-2026-067.

### 2.2 Instruments

#### 2.2.1 Sedentary behavior

Sedentary behavior was assessed using the Sedentary Behavior Questionnaire (SBQ), originally developed by Rosenberg (16). to evaluate time spent in common sedentary activities across weekdays and weekend days. The SBQ has been used to assess multiple sedentary contexts, including television viewing, computer use, reading, transportation, and other sitting-based activities. In the present study, the questionnaire was adapted to the university context and included nine common sedentary domains: studying while seated, using a computer, watching television or online videos, playing video games, using a smartphone, reading, sitting during transportation, socializing while seated, and other sedentary leisure activities.

Participants reported the average time spent in each sedentary activity on weekdays and weekend days during the past week. Example items included “time spent studying while seated,” “time spent using a smartphone while sitting,” and “time spent watching television or online videos while seated.” For each activity, weekday and weekend sedentary time were first calculated separately. Average daily sedentary time was then computed using the following weighted formula: Average daily sedentary time = [(weekday sedentary time × 5) + (weekend sedentary time × 2)] / 7.

Higher scores indicated longer daily sedentary time. In the present study, total average daily sedentary time was used as the main indicator of sedentary behavior. The internal consistency of the SBQ in this sample was acceptable, with Cronbach's α values of 0.678 at T1, 0.648 at T2, and 0.664 at T3.

#### 2.2.2 Body image

Body image was measured using a 20-item multidimensional body image scale adapted from the appearance-related domains of the Multidimensional Body-Self Relations Questionnaire Appearance Scales (BI) (17, 18). The BI is a widely used instrument for assessing appearance-related body image and includes domains such as appearance evaluation, appearance orientation, body-area satisfaction, overweight preoccupation, and self-classified weight. In the present study, the measure was used to assess students' perceptions, evaluations, and affective attitudes toward their physical appearance and body-related self-perception.

Items were rated on a 5-point Likert scale ranging from 1 = strongly disagree to 5 = strongly agree. Example items included statements reflecting satisfaction with one's appearance, perceived attractiveness, concern about body shape, and satisfaction with specific body areas. Negatively worded items were reverse-coded so that higher scores indicated a more positive body image. The mean score of all items was calculated, with higher scores reflecting more favorable body-related self-evaluation.

The scale demonstrated excellent internal consistency across the three waves. Cronbach's α values were 0.943 at T1, 0.945 at T2, and 0.943 at T3..

#### 2.2.3 Physical activity enjoyment

Physical activity enjoyment was assessed using an 8-item version of the Physical Activity Enjoyment Scale (PACES). The original PACES was developed by Kendzierski and DeCarlo to assess the extent to which individuals experience enjoyment during physical activity, and short forms of PACES have been widely used in exercise and health behavior research (11). In this study, the scale was used to measure students' positive affective experience when engaging in physical activity or exercise.

Participants were instructed to respond according to their general experience of physical activity during the past month. For students who rarely participated in structured exercise, they were asked to answer based on their most recent experience of physical activity, including informal exercise, recreational sports, walking, or other movement-based activities. Items were rated on a 5-point Likert scale ranging from 1 = strongly disagree to 5 = strongly agree. Example items assessed whether participants found physical activity enjoyable, pleasant, interesting, or satisfying. Negatively worded items were reverse-coded when necessary, and higher mean scores indicated greater physical activity enjoyment.

The scale showed good internal consistency in the present sample, with Cronbach's α values of 0.893 at T1, 0.893 at T2, and 0.899 at T3.

#### 2.2.4 Life satisfaction

Life satisfaction was assessed using the Satisfaction With Life Scale (SWLS), developed by Diener, Emmons, Larsen, and Griffin (4). The SWLS is a five-item scale designed to measure individuals' global cognitive evaluation of their life satisfaction rather than positive or negative affect. In the present study, the SWLS was administered at all three waves to support longitudinal measurement invariance testing, cross-lagged panel analysis, and the longitudinal mediation model.

Participants rated each item on a 7-point Likert scale ranging from 1 = strongly disagree to 7 = strongly agree. Example items included statements assessing whether participants regarded their life as close to ideal, were satisfied with their life, and believed that their life conditions were good. The total score was calculated by summing the five items, with higher scores indicating higher life satisfaction. The possible total score ranged from 5 to 35.

The SWLS showed good internal consistency across the three waves, with Cronbach's α values of 0.870 at T1, 0.881 at T2, and 0.867 at T3.

### 2.3 Data analysis

All statistical analyses were conducted using SPSS (SPSS 25.0) and AMOS (Amos 23.0). Descriptive statistics, reliability analyses, and correlations were based on observed scale scores. Confirmatory factor analysis (CFA) and longitudinal measurement models were estimated by maximum likelihood. Latent factors were identified using a marker-variable approach in which the first loading of each factor was fixed to 1.0, with factor variances and covariances freely estimated. Statistical tests were two-tailed with p < .05. Overall model fit was evaluated jointly using χ^2^, CFI, TLI, RMSEA, and SRMR, following commonly used recommendations (19).

First, raw data were screened for invalid or implausible values before score computation. The inferential analytic dataset comprised 426 participants with complete matched T1–T3 data and no item-level missing values. Consequently, the longitudinal models were estimated using these complete cases. Although participant-flow records indicated larger samples at T1 and T2, the analysis file did not retain the wave-specific records of participants who did not complete T3; FIML using those unavailable observations could therefore not be implemented retrospectively. The implications of complete-case analysis are acknowledged in the Limitations section.

Second, participant retention was summarized from the study flow records (526 at T1, 476 at T2, and 426 at T3). Because noncompleter records were not retained in the inferential analytic file, baseline comparisons between retained and attrited participants could not be reconstructed. The resulting possibility of attrition bias is therefore treated explicitly as a study limitation rather than inferred from unavailable data.

Third, descriptive statistics and Pearson correlation coefficients were calculated for the main study variables, including sedentary behavior, body image, physical activity enjoyment, life satisfaction, physical activity level, BMI, and demographic characteristics. Internal consistency was summarized using Cronbach's alpha and composite reliability indices where applicable.

Fourth, CFA was used to evaluate the measurement structure of body image (BI), physical activity enjoyment (PACES), and life satisfaction (SWLS). BI and PACES were each represented by four parcels, whereas the five SWLS items were retained as separate indicators, yielding 13 observed indicators in the three-factor model. Parceling followed the item-to-construct balance approach (20) using standardized one-factor loadings at T1, and the same parcel composition was retained across all three waves. BI parcels were P1 = items 16, 12, 3, 17, and 4; P2 = items 19, 14, 20, 1, and 7; P3 = items 9, 6, 10, 8, and 2; and P4 = items 5, 18, 11, 15, and 13. PACES parcels were P1 = items 3 and 2; P2 = items 8 and 7; P3 = items 4 and 6; and P4 = items 1 and 5. Parcel scores were item means. The hypothesized three-factor model was compared with a one-factor alternative, and standardized factor loadings are reported in Supplementary Table S1. Model fit was assessed using χ^2^/df, CFI, TLI, RMSEA, and SRMR (19).

Fifth, longitudinal measurement invariance was evaluated separately for BI, PACES, and SWLS. Configural models retained the same measurement pattern across waves; metric models constrained corresponding factor loadings to equality; and scalar models additionally constrained corresponding intercepts. Changes in fit were evaluated using ΔCFI ≤ .010 and ΔRMSEA ≤ .015 as complementary benchmarks (21, 22). When full invariance was not supported, minimal partial-invariance models were examined while retaining sufficient invariant indicators for factor identification. For BI, partial scalar invariance was obtained by releasing the intercept equality constraints for BI-P3 and BI-P4. For PACES, partial metric invariance was obtained by releasing the loading equality constraints for PACES-P2 and PACES-P4; scalar invariance was not supported. For SWLS, partial metric invariance was obtained by releasing the SWLS4 loading constraint, and partial scalar invariance was obtained by retaining equal intercepts for SWLS1 and SWLS4 while releasing those for SWLS2, SWLS3, and SWLS5.

Sixth, common method variance was examined using Harman's single-factor diagnostic and comparison of the hypothesized measurement model with a one-factor CFA. These procedures were treated as limited diagnostics rather than as tests capable of establishing the absence of common method bias (23). The longitudinal temporal separation of measurements also reduced, but did not eliminate, the possibility of shared-method inflation.

Seventh, a fully reciprocal manifest-score CLPM was estimated using observed scale scores for sedentary behavior, BI, PACES, and SWLS; measurement error was therefore not modeled in the structural panel. Each T2 construct was regressed on all four T1 constructs, and each T3 construct was regressed on all four T2 constructs. Thus, all autoregressive and cross-lagged paths were estimated for every construct pair in both adjacent intervals, with within-wave covariances or residual covariances freely estimated. All paths, including nonsignificant paths, are reported in Table 1 and summarized in Figure 1. Because a traditional CLPM does not separate stable between-person differences from within-person fluctuations, coefficients were interpreted as prospective between-person associations rather than within-person effects (24). A full-forward robustness model additionally regressed every T3 construct on all four T1 constructs, thereby adding the complete set of T1 → T3 lag-2 paths (25–27).

**Figure 1:**
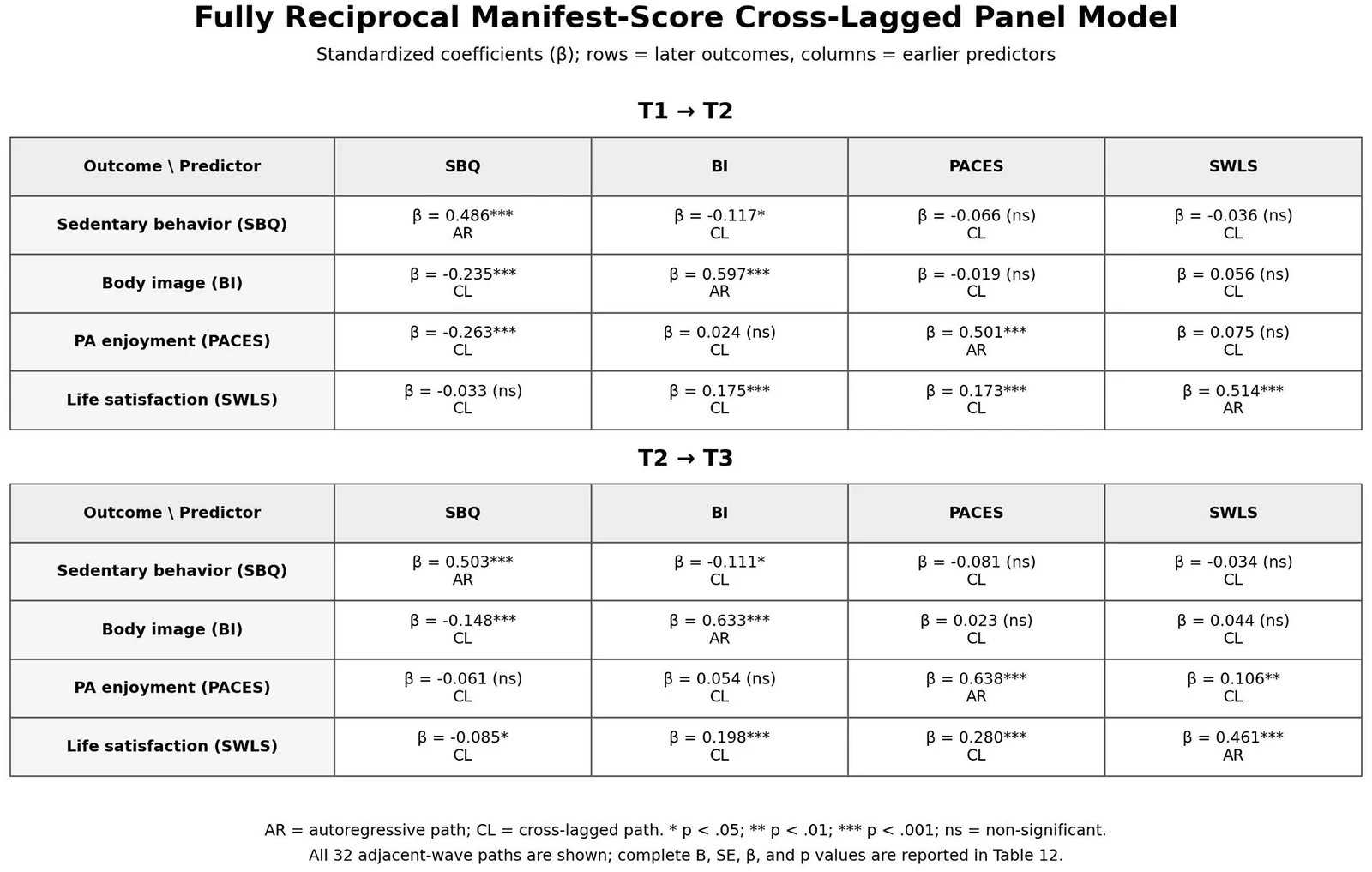
Fully Reciprocal Manifest-Score CLPM standardized coefficient matrices.

**Table 1:** All autoregressive and cross-lagged paths in the fully reciprocal manifest-score CLPM.

Lag	Dependent	Predictor	Type	*B*	SE	β	*p*
T1 → T2	SBQ	SBQ	Autoregressive	0.480	0.044	0.486	< 0.001
T1 → T2	SBQ	BI	Cross-lagged	−0.216	0.085	−0.117	0.011
T1 → T2	SBQ	PACES	Cross-lagged	−0.112	0.078	−0.066	0.155
T1 → T2	SBQ	SWLS	Cross-lagged	−0.008	0.011	−0.036	0.464
T1 → T2	BI	SBQ	Cross-lagged	−0.127	0.020	−0.235	< 0.001
T1 → T2	BI	BI	Autoregressive	0.602	0.038	0.597	< 0.001
T1 → T2	BI	PACES	Cross-lagged	−0.017	0.035	−0.019	0.621
T1 → T2	BI	SWLS	Cross-lagged	0.007	0.005	0.056	0.163
T1 → T2	PACES	SBQ	Cross-lagged	−0.151	0.023	−0.263	< 0.001
T1 → T2	PACES	BI	Cross-lagged	0.026	0.044	0.024	0.556
T1 → T2	PACES	PACES	Autoregressive	0.496	0.041	0.501	< 0.001
T1 → T2	PACES	SWLS	Cross-lagged	0.010	0.006	0.075	0.085
T1 → T2	SWLS	SBQ	Cross-lagged	−0.147	0.172	−0.033	0.394
T1 → T2	SWLS	BI	Cross-lagged	1.454	0.328	0.175	< 0.001
T1 → T2	SWLS	PACES	Cross-lagged	1.328	0.303	0.173	< 0.001
T1 → T2	SWLS	SWLS	Autoregressive	0.518	0.042	0.514	< 0.001
T2 → T3	SBQ	SBQ	Autoregressive	0.516	0.045	0.503	< 0.001
T2 → T3	SBQ	BI	Cross-lagged	−0.208	0.082	−0.111	0.012
T2 → T3	SBQ	PACES	Cross-lagged	−0.143	0.080	−0.081	0.074
T2 → T3	SBQ	SWLS	Cross-lagged	−0.008	0.010	−0.034	0.452
T2 → T3	BI	SBQ	Cross-lagged	−0.080	0.020	−0.148	< 0.001
T2 → T3	BI	BI	Autoregressive	0.624	0.037	0.633	< 0.001
T2 → T3	BI	PACES	Cross-lagged	0.021	0.036	0.023	0.559
T2 → T3	BI	SWLS	Cross-lagged	0.005	0.005	0.044	0.260
T2 → T3	PACES	SBQ	Cross-lagged	−0.037	0.022	−0.061	0.099
T2 → T3	PACES	BI	Cross-lagged	0.059	0.041	0.054	0.146
T2 → T3	PACES	PACES	Autoregressive	0.653	0.039	0.638	< 0.001
T2 → T3	PACES	SWLS	Cross-lagged	0.014	0.005	0.106	0.005
T2 → T3	SWLS	SBQ	Cross-lagged	−0.377	0.154	−0.085	0.015
T2 → T3	SWLS	BI	Cross-lagged	1.598	0.283	0.198	< 0.001
T2 → T3	SWLS	PACES	Cross-lagged	2.126	0.274	0.280	< 0.001
T2 → T3	SWLS	SWLS	Autoregressive	0.452	0.035	0.461	< 0.001

Eighth, the longitudinal parallel mediation model was estimated as an observed-variable path model. T1 sedentary behavior was specified as the predictor, T2 BI and T2 PACES as parallel mediators, and T3 SWLS as the outcome. T1 BI and T1 PACES were included as prior levels of the mediators. The outcome equation included both T2 SWLS, representing the immediately preceding outcome level, and T1 SWLS, thereby incorporating the adjacent T2 SWLS → T3 SWLS autoregressive path recommended for longitudinal mediation analyses (28). Primary models adjusted for gender, age, BMI, MVPA min/week, and physical activity level; broader sociodemographic adjustment was examined as a sensitivity analysis.

Indirect effects through BI and PACES were estimated using 5,000 bootstrap resamples with bias-corrected 95% confidence intervals. An indirect effect was considered statistically distinguishable from zero when its interval excluded zero. The difference between the two specific indirect effects was also bootstrapped. Because T2 SWLS was treated as an immediate prior-outcome control rather than a focal mediator, the sum of the BI and PACES indirect effects is described as the combined focal indirect effect rather than as the total causal effect.

## 3 Results

### 3.1 Sample description

Table 2 summarizes the sociodemographic characteristics of the study sample. A total of 426 participants were included. The mean age was 20.54 years (SD = 1.57), mean weight was 61.08 kg (SD = 10.10), and mean body mass index was 21.75 kg/m^2^ (SD = 2.73). Of the participants, 238 (55.9%) were female and 188 (44.1%) were male. Regarding education level, 327 participants (76.8%) were undergraduate students, 59 (13.8%) were master's students, and 40 (9.4%) were doctoral students. The distribution by grade was as follows: 79 freshmen (18.5%), 91 sophomores (21.4%), 83 juniors (19.5%), 74 seniors (17.4%), 59 master's students (13.8%), and 40 doctoral students (9.4%). In terms of major category, 96 participants (22.5%) were in humanities, 96 (22.5%) in social sciences, 82 (19.2%) in science/engineering, 81 (19.0%) in medical/health, and 71 (16.7%) in sports/arts.

**Table 2:** Sociodemographic characteristics.

Variable	Category	*n* (%) or M ± SD
Age, years	—	20.54 ± 1.57
Weight, kg	—	61.08 ± 10.10
BMI, kg/m^2^	—	21.75 ± 2.73
Gender	Male	188 (44.1)
	Female	238 (55.9)
Education level	Undergraduate	327 (76.8)
	Master's student	59 (13.8)
	Doctoral student	40 (9.4)
Grade	Freshman	79 (18.5)
	Sophomore	91 (21.4)
	Junior	83 (19.5)
	Senior	74 (17.4)
	Master's student	59 (13.8)
	Doctoral student	40 (9.4)
Major category	Humanities	96 (22.5)
	Social sciences	96 (22.5)
	Science/engineering	82 (19.2)
	Medical/health	81 (19.0)
	Sports/arts	71 (16.7)

As shown in Table 3, the final analytic sample included 426 participants. The mean sedentary behavior was 8.188 h/day at T1, 8.189 h/day at T2, and 8.133 h/day at T3. Body image scores were 3.067 ± 0.822 at T1, 2.961 ± 0.828 at T2, and 3.084 ± 0.817 at T3. Physical activity enjoyment scores were 3.059 ± 0.891 at T1, 2.962 ± 0.882 at T2, and 3.048 ± 0.904 at T3. Life satisfaction scores were 20.021 ± 6.782 at T1, 21.631 ± 6.833 at T2, and 20.613 ± 6.700 at T3. The mean BMI was 21.751 ± 2.735 kg/m^2^, and the mean moderate-to-vigorous physical activity was 123.171 ± 80.203 min/week. Skewness and kurtosis values for most study variables were within an acceptable range. The only variable showing relatively high skewness and kurtosis was MVPA, with a skewness value of 2.114 and a kurtosis value of 7.808.

**Table 3:** Descriptive statistics of study variables.

Variable	*N*	Mean	SD	Min	Max	Skewness	Kurtosis
T1 Sedentary behavior (h/day)	426	8.188	1.536	3.600	13.460	0.086	0.154
T2 Sedentary behavior (h/day)	426	8.189	1.518	3.830	12.410	−0.095	0.096
T3 Sedentary behavior (h/day)	426	8.133	1.556	4.100	13.090	0.209	−0.106
T1 Body image	426	3.067	0.822	1.200	4.950	−0.017	−0.767
T2 Body image	426	2.961	0.828	1.150	4.950	0.119	−0.690
T3 Body image	426	3.084	0.817	1.200	4.950	0.032	−0.591
T1 Physical activity enjoyment	426	3.059	0.891	1.000	5.000	−0.028	−0.580
T2 Physical activity enjoyment	426	2.962	0.882	1.000	5.000	0.100	−0.718
T3 Physical activity enjoyment	426	3.048	0.904	1.000	5.000	0.127	−0.606
T1 Life satisfaction	426	20.021	6.782	5.000	35.000	−0.021	−0.579
T2 Life satisfaction	426	21.631	6.833	6.000	35.000	−0.174	−0.693
T3 Life satisfaction	426	20.613	6.700	5.000	35.000	0.033	−0.842
BMI	426	21.751	2.735	16.480	29.070	0.122	−0.335
MVPA (min/week)	426	123.171	80.203	20.000	685.000	2.114	7.808

### 3.2 Reliability analyses

Table 4 presents the internal consistency estimates for all scales across the three waves. Cronbach's alpha values for body image ranged from 0.943 to 0.945. For physical activity enjoyment, Cronbach's alpha ranged from 0.893 to 0.899. For life satisfaction, Cronbach's alpha ranged from 0.867 to 0.881. The sedentary behavior measure showed lower Cronbach's alpha values, ranging from 0.648 to 0.678. Composite reliability values for body image, physical activity enjoyment, and life satisfaction were all above 0.90. The AVE values ranged from 0.483 to 0.491 for body image, from 0.573 to 0.589 for physical activity enjoyment, and from 0.654 to 0.679 for life satisfaction.

**Table 4:** Reliability analyses.

Scale	Time	Items	Cronbach α	CR	AVE
Sedentary behavior	T1	9	0.678	0.774	0.287
Body image	T1	20	0.943	0.949	0.485
Physical activity enjoyment	T1	8	0.893	0.915	0.574
Life satisfaction	T1	5	0.870	0.907	0.661
Sedentary behavior	T2	9	0.648	0.765	0.272
Body image	T2	20	0.945	0.950	0.491
Physical activity enjoyment	T2	8	0.893	0.914	0.573
Life satisfaction	T2	5	0.881	0.913	0.679
Sedentary behavior	T3	9	0.664	0.772	0.281
Body image	T3	20	0.943	0.949	0.483
Physical activity enjoyment	T3	8	0.899	0.919	0.589
Life satisfaction	T3	5	0.867	0.904	0.654

### 3.3 Correlation analyses

Table 5 and Figure 2 shows the Pearson correlations among the main study variables. T1 sedentary behavior was negatively correlated with T1 body image, T1 physical activity enjoyment, T1 life satisfaction, T2 body image, T2 physical activity enjoyment, and T3 life satisfaction. The correlation between T1 sedentary behavior and T3 life satisfaction was significant and negative, *r* = −0.463, *p* < 0.001. T2 body image was positively correlated with T3 life satisfaction, *r* = 0.479, *p* < 0.001. T2 physical activity enjoyment was also positively correlated with T3 life satisfaction, *r* = 0.561, *p* < 0.001. T1 life satisfaction was positively associated with T3 life satisfaction, *r* = 0.611, *p* < 0.001. The correlation between T2 body image and T2 physical activity enjoyment was also significant, *r* = 0.287, *p* < 0.001.

**Table 5:** Pearson correlations among main variables.

Variable	1	2	3	4	5	6	7
1. T1 SBQ	—						
2. T1 BI	−0.385^***^	—					
3. T1 PACES	−0.333^***^	0.169^***^	—				
4. T1 SWLS	−0.314^***^	0.420^***^	0.457^***^	—			
5. T2 BI	−0.476^***^	0.707^***^	0.186^***^	0.372^***^	—		
6. T2 PACES	−0.463^***^	0.242^***^	0.627^***^	0.397^***^	0.287^***^	—	
7. T3 SWLS	−0.463^***^	0.435^***^	0.525^***^	0.611^***^	0.479^***^	0.561^***^	—

SBQ, sedentary behavior; BI, body image; PACES, physical activity enjoyment; SWLS, life satisfaction.

^***^p < 0.001.

**Figure 2:**
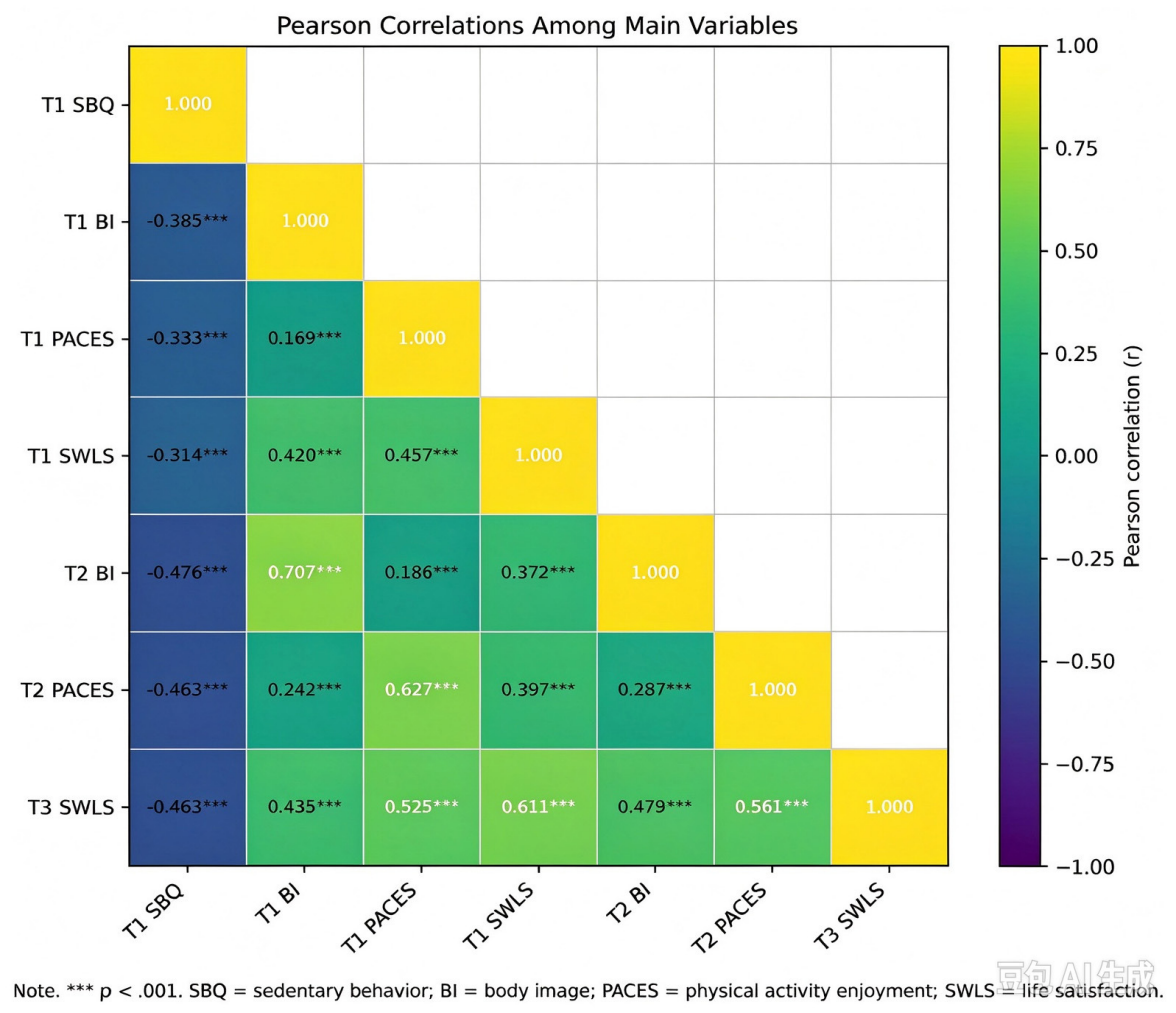
Heatmap of correlations among key variables.

### 3.4 Missing data and attrition analyses

The participant-flow profile is presented in Table 6. Study records indicated 526 valid participants at T1, 476 at T2, and 426 at T3, corresponding to an overall retention rate of 81.0%. The inferential analytic dataset contained the 426 matched T1–T3 completers and no item-level missing values. Wave-specific records for the 100 participants who did not complete all waves were not retained in this analysis file; therefore, FIML using those observations and retained-vs.-attrited baseline comparisons could not be performed from the archived inferential dataset. All inferential analyses below use the 426 complete longitudinal cases.

**Table 6:** Missing data and attrition analyses.

Assessment wave	Time point	Valid participants, *n*	Dropout since previous wave*, n* (%)	Retention from baseline, %	Item-level missingness in analytic dataset
Baseline survey	T1	526	—	100.0	Not available for noncompleter records
First follow-up	T2, 6 months	476	50 (9.5)	90.5	Not available for noncompleter records
Second follow-up	T3, 12 months	426	50 (10.5)	81.0	0.00% among retained completers
Final analytic sample	T1–T3 complete cases	426	100 (19.0)	81.0	0.00%

### 3.5 Confirmatory factor analysis

Table 7 presents the CFA results for the explicitly documented 13-indicator structure. The three-factor model showed close fit at all waves: T1, χ^2^ = 48.019, df = 62, CFI = 1.000, TLI = 1.000, RMSEA = 0.000, SRMR = 0.016; T2, χ^2^ = 75.369, df = 62, CFI = 0.997, TLI = 0.996, RMSEA = 0.023, SRMR = 0.026; and T3, χ^2^ = 52.658, df = 62, CFI = 1.000, TLI = 1.000, RMSEA = 0.000, SRMR = 0.019. Standardized factor loadings ranged from 0.668 to 0.911 across waves. The one-factor models fit substantially worse at all waves, supporting the distinction among BI, PACES, and SWLS. At T1 and T3, χ^2^ was smaller than df, producing a non-positive noncentrality estimate; accordingly, RMSEA was bounded at 0.000 and CFI/TLI reached their upper bound. These boundary values indicate very close sample fit rather than error-free measurement.

**Table 7:** Measurement model fit indices.

Model	χ^2^	df	χ^2^/df	CFI	TLI	RMSEA	SRMR
T1 three-factor model	48.019	62	0.775	1.000	1.000	0.000	0.016
T2 three-factor model	75.369	62	1.216	0.997	0.996	0.023	0.026
T3 three-factor model	52.658	62	0.849	1.000	1.000	0.000	0.019
T1 one-factor model	1879.950	65	28.922	0.514	0.416	0.256	0.240
T2 one-factor model	1911.580	65	29.409	0.520	0.424	0.259	0.229
T3 one-factor model	1720.428	65	26.468	0.558	0.470	0.245	0.213

### 3.6 Longitudinal measurement invariance

As shown in Table 8, BI demonstrated configural and full metric invariance. Full scalar invariance was not supported, whereas partial scalar invariance was supported after releasing the intercept constraints for BI-P3 and BI-P4 (relative to the metric model: ΔCFI = −0.002, ΔRMSEA = 0.014). PACES did not satisfy the full metric criterion because ΔRMSEA = 0.037, although ΔCFI = −0.009; partial metric invariance was supported after releasing the loading equality constraints for PACES-P2 and PACES-P4 (ΔCFI = 0.000, ΔRMSEA = 0.000), but scalar invariance was not supported. For SWLS, the full metric model exceeded the ΔRMSEA benchmark (0.022); partial metric invariance was supported after releasing the SWLS4 loading equality constraint (ΔCFI = −0.002, ΔRMSEA = 0.015). Full scalar invariance was not supported, whereas partial scalar invariance was supported when SWLS1 and SWLS4 intercepts were held equal and SWLS2, SWLS3, and SWLS5 intercepts were released (relative to the partial metric model: ΔCFI = −0.007, ΔRMSEA = 0.014). Thus, longitudinal structural associations can be interpreted with appropriate caution, whereas latent mean comparisons are not supported for PACES.

**Table 8:** Longitudinal measurement invariance.

Construct	Model	*χ^2^*	df	χ^2^/df	CFI	TLI	RMSEA	SRMR	ΔCFI	ΔRMSEA
BI	Configural	49.674	51	0.974	1.000	1.000	0.000	0.015	—	—
BI	Metric	58.055	57	1.019	1.000	1.000	0.007	0.027	−0.000	0.007
BI	Scalar	162.126	63	2.573	0.981	0.980	0.061	0.027	−0.018	0.054
BI	Partial scalar	69.184	59	1.173	0.998	0.998	0.020	0.027	−0.002	0.014
PACES	Configural	47.639	51	0.934	1.000	1.000	0.000	0.018	—	—
PACES	Metric	90.190	57	1.582	0.991	0.989	0.037	0.056	−0.009	0.037
PACES	Partial metric	50.259	53	0.948	1.000	1.000	0.000	0.022	0.000	0.000
PACES	Scalar (after partial metric)	303.131	59	5.138	0.931	0.923	0.099	0.042	−0.069	0.099
SWLS	Configural	76.677	87	0.881	1.000	1.000	0.000	0.021	—	—
SWLS	Metric	114.541	95	1.206	0.995	0.994	0.022	0.055	−0.005	0.022
SWLS	Partial metric	101.500	93	1.091	0.998	0.997	0.015	0.047	−0.002	0.015
SWLS	Scalar (after partial metric)	657.526	101	6.510	0.843	0.837	0.114	0.059	−0.154	0.099
SWLS	Partial scalar	128.305	95	1.351	0.991	0.990	0.029	0.049	−0.007	0.014

ΔCFI and ΔRMSEA compare each constrained model with the stated less-constrained reference: metric/partial metric versus configural, and scalar/partial scalar vs. the accepted metric or partial-metric model. PACES-P2 and PACES-P4 loading equalities were released for partial metric invariance; SWLS4 loading equality was released for SWLS partial metric invariance. BI-P3/BI-P4 intercepts and SWLS2/SWLS3/SWLS5 intercepts were released in the corresponding partial-scalar models.

### 3.7 Common method bias

Table 9 presents limited diagnostics related to common method variance. Harman's first unrotated factor explained 33.139% of the variance. In the parcel-based CFA, the T1 one-factor model fit poorly (CFI = 0.514, RMSEA = 0.256, SRMR = 0.240), whereas the hypothesized three-factor model fit closely (CFI = 1.000, RMSEA = 0.000, SRMR = 0.016). These findings indicate that a single common factor does not adequately reproduce the observed covariance structure, but they do not establish the absence of common method bias.

**Table 9:** Common method bias test.

Test	Index	Value	Criterion	Conclusion
Harman single-factor diagnostic	Variance explained by first factor (%)	33.139	Descriptive diagnostic	No dominant single factor detected; CMB cannot be ruled out
Single-factor CFA (T1)	CFI/RMSEA/SRMR	0.514/0.256/0.240	Poor fit	Single-factor representation inadequate
Hypothesized three-factor CFA (T1)	CFI/RMSEA/SRMR	1.000/0.000/0.016	Close fit	Supports construct distinctiveness; not a definitive CMB test

CMB, common method bias.

### 3.8 Structural model testing

The longitudinal mediation/path model is presented in Table 10. T1 sedentary behavior negatively predicted T2 body image (*B* = −0.122, β = −0.227, *p* < 0.001) and T2 physical activity enjoyment (*B* = −0.142, β = −0.248, *p* < 0.001), while prior BI and PACES showed strong autoregressive associations with their T2 scores. In the T3 life-satisfaction equation, which included both T2 and T1 SWLS as prior-outcome controls, T1 sedentary behavior remained negatively associated with T3 SWLS (*B* = −0.414, β = −0.095, *p* = 0.013). T2 BI (*B* = 1.223, β = 0.151, *p* < 0.001) and T2 PACES (*B* = 1.669, β = 0.220, *p* < 0.001) independently predicted higher T3 SWLS. The adjacent T2 SWLS → T3 SWLS path was significant (*B* = 0.342, β = 0.349, *p* < 0.001), and T1 SWLS retained a smaller independent association (*B* = 0.170, β = 0.172, *p* < 0.001).

**Table 10:** Structural path coefficients.

Dependent	Predictor	*B*	SE	β	*t*	*p*	95% CI
T2 Body image	T1 SBQ	−0.122	0.020	−0.227	−6.161	< 0.001	[−0.161, −0.083]
T2 Body image	T1 BI	0.571	0.038	0.567	15.171	< 0.001	[0.498, 0.645]
T2 PACES	T1 SBQ	−0.142	0.022	−0.248	−6.460	< 0.001	[−0.185, −0.099]
T2 PACES	T1 PACES	0.465	0.039	0.469	11.915	< 0.001	[0.388, 0.541]
T3 SWLS	T1 SBQ	−0.414	0.166	−0.095	−2.486	0.013	[−0.740, −0.088]
T3 SWLS	T2 BI	1.223	0.308	0.151	3.963	< 0.001	[0.618, 1.827]
T3 SWLS	T2 PACES	1.669	0.290	0.220	5.759	< 0.001	[1.101, 2.237]
T3 SWLS	T2 SWLS	0.342	0.042	0.349	8.121	< 0.001	[0.259, 0.424]
T3 SWLS	T1 SWLS	0.170	0.042	0.172	4.061	< 0.001	[0.088, 0.253]

Table 11 and Figure 3 present the bootstrap mediation results after inclusion of T2 SWLS. The indirect effect through body image was significant [*b* = −0.150, Boot SE = 0.042, bias-corrected 95% CI (−0.247, −0.079)], as was the indirect effect through physical activity enjoyment [*b* = −0.237, Boot SE = 0.057, 95% CI (−0.368, −0.141)]. The combined focal indirect effect was −0.387 [Boot SE = 0.071, 95% CI (−0.543, −0.259)]. The direct effect of T1 sedentary behavior on T3 life satisfaction remained significant [b = −0.414, Boot SE = 0.161, 95% CI (−0.742, −0.095)]. These results indicate partial mediation after adjustment for the immediately preceding outcome level.

**Table 11:** Parallel mediation effects.

Effect	Estimate	Boot SE	95% CI Lower	95% CI Upper	Significant
Indirect via BI	−0.150	0.042	−0.247	−0.079	Yes
Indirect via PACES	−0.237	0.057	−0.368	−0.141	Yes
Combined focal indirect	−0.387	0.071	−0.543	−0.259	Yes
Direct effect	−0.414	0.161	−0.742	−0.095	Yes

Indirect effects were estimated using 5,000 bootstrap resamples. Coefficients are unstandardized.

**Figure 3:**
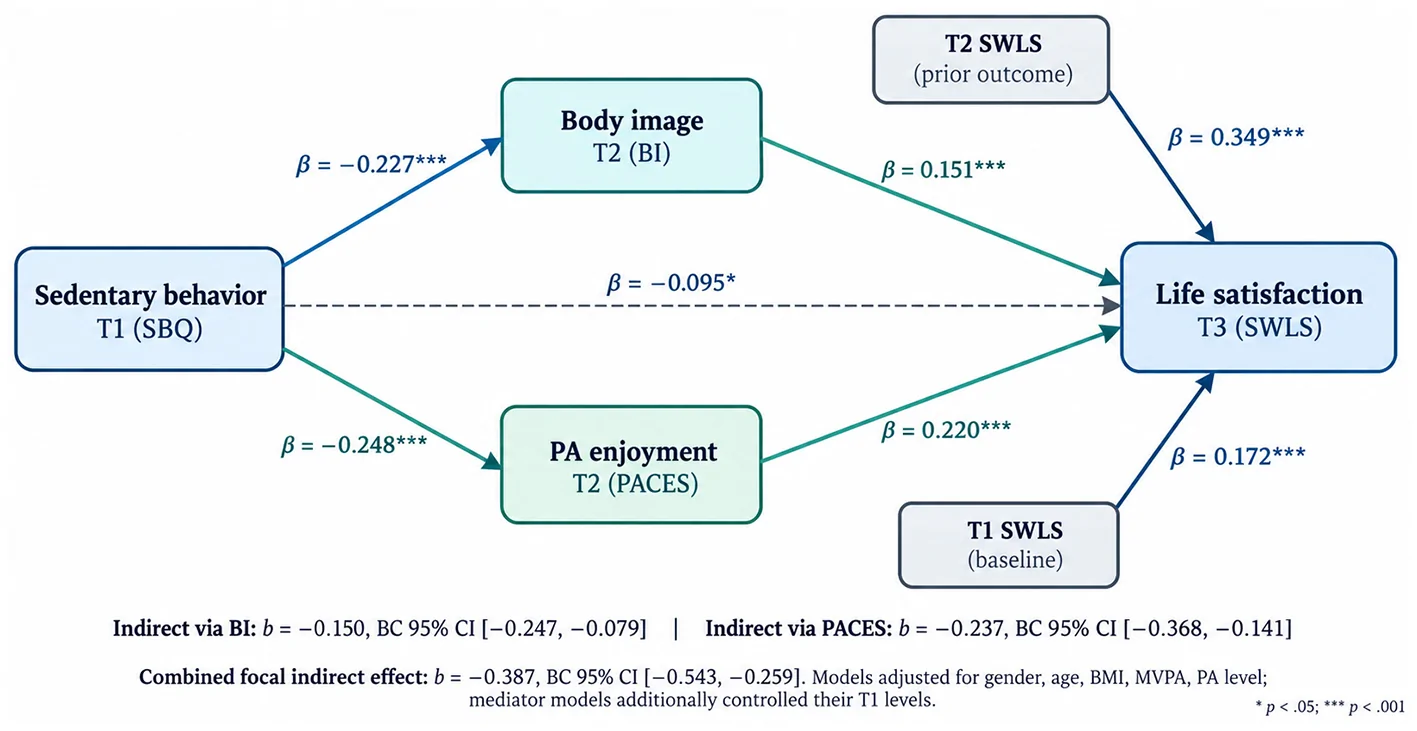
Longitudinal parallel mediation model controlling T2 and T1 life satisfaction.

As shown in Table 12, the difference between the two specific indirect effects was not statistically significant [difference = 0.088, Boot SE = 0.070, bias-corrected 95% CI (−0.040, 0.237)]. Of the combined focal indirect effect, the body-image pathway accounted for 38.6% and the physical-activity-enjoyment pathway for 61.4%. The two pathways should therefore be interpreted as complementary rather than statistically different in strength.

**Table 12:** Comparative strength of mediators.

Effect	Estimate	Boot SE	95% CI Lower	95% CI Upper	Significant	Mediator	Indirect effect	Share of combined focal indirect (%)
BI indirect–PACES indirect	0.088	0.070	−0.040	0.237	No	—	—	—
—	—	—	—	—	—	Body image	−0.150	38.6
—	—	—	—	—	—	Physical activity enjoyment	−0.237	61.4

### 3.9 Fully reciprocal manifest-score cross-lagged panel analysis

The fully reciprocal manifest-score CLPM showed acceptable overall fit, χ^2^ = 45.397, df = 16, χ^2^/df = 2.837, CFI = 0.989, TLI = 0.954, RMSEA = 0.066, and SRMR = 0.020. Table 1 and Figure 1 reports all 32 adjacent-wave autoregressive and cross-lagged paths, irrespective of statistical significance. Autoregressive stability was moderate to strong across all constructs. Importantly, the reciprocal specification also identified several reverse prospective paths, including BI → later SBQ at both intervals (T1 → T2: β = −0.117, *p* 0011; T2 → T3: β = −0.111, *p* = 0.012), while PACES → later SBQ and SWLS → later SBQ were not significant.

From T1 → T2, T1 sedentary behavior predicted lower T2 BI (β = −0.235, *p* < 0.001) and lower T2 PACES (β = −0.263, *p* < 0.001), but did not directly predict T2 SWLS after all four T1 constructs were entered simultaneously (β = −0.033, *p* = 0.394). T1 BI (β = 0.175, *p* < 0.001) and T1 PACES (β = 0.173, *p* < 0.001) independently predicted higher T2 SWLS. Other cross-lagged paths are reported in Table 1.

From T2 → T3, T2 sedentary behavior predicted lower T3 BI (β = −0.148, *p* < 0.001) and lower T3 SWLS (β = −0.085, *p* = 0.015), while its path to T3 PACES was not statistically significant (β = −0.061, *p* = 0.099). T2 BI (β = 0.198, *p* < 0.001) and T2 PACES (β = 0.280, *p* < 0.001) predicted higher T3 SWLS. T2 SWLS also showed a small positive cross-lagged association with T3 PACES (β = 0.106, *p* = 0.005). These coefficients describe prospective between-person associations in observed scores and should not be interpreted as within-person causal effects.

As a robustness analysis, a full-forward CLPM was estimated by adding all T1 → T3 lag-2 paths. With three waves and freely estimated within-wave residual covariances, this specification is saturated (df = 0), so global fit indices are not diagnostically informative. The focal T2 → T3 mediator-to-outcome paths remained significant after the lag-2 paths were added (BI → SWLS: β = 0.176, *p* < 0.001; PACES → SWLS: β = 0.182, *p* < 0.001). The T2 SBQ → T3 BI association also remained significant (β = −0.118, *p* = 0.005), whereas T2 SBQ → T3 SWLS was attenuated and no longer significant (β = −0.050, *p* = 0.180). Table 13 reports all T1 → T3 lag-2 coefficients from the full-forward model. This pattern supports the robustness of the mediator-to-outcome pathways but warrants caution in interpreting a direct adjacent-wave SBQ → SWLS effect.

**Table 13:** T1 → T3 Lag-2 paths in the full-forward robustness model.

Dependent	Predictor	*B*	SE	β	*p*
T3 SBQ	T1 SBQ	0.082	0.053	0.081	0.124
T3 SBQ	T1 BI	−0.170	0.109	−0.090	0.119
T3 SBQ	T1 PACES	−0.011	0.092	−0.007	0.902
T3 SBQ	T1 SWLS	−0.023	0.013	−0.100	0.073
T3 BI	T1 SBQ	−0.031	0.024	−0.058	0.201
T3 BI	T1 BI	0.078	0.050	0.078	0.117
T3 BI	T1 PACES	0.047	0.042	0.051	0.266
T3 BI	T1 SWLS	0.003	0.006	0.025	0.609
T3 PACES	T1 SBQ	0.022	0.026	0.038	0.397
T3 PACES	T1 BI	0.012	0.054	0.011	0.826
T3 PACES	T1 PACES	0.053	0.046	0.052	0.251
T3 PACES	T1 SWLS	0.007	0.006	0.052	0.275
T3 SWLS	T1 SBQ	−0.321	0.178	−0.074	0.072
T3 SWLS	T1 BI	−0.101	0.365	−0.012	0.783
T3 SWLS	T1 PACES	0.880	0.309	0.117	0.005
T3 SWLS	T1 SWLS	0.155	0.043	0.157	< 0.001

### 3.10 Sensitivity analyses

The sensitivity analyses are shown in Table 14. The primary specification yielded a direct effect of −0.414, a BI indirect effect of −0.150, a PACES indirect effect of −0.237, and a combined focal indirect effect of −0.387. The pattern remained substantively similar after removing MVPA/PA-level covariates, excluding T1 SBQ outliers beyond ±3 SD, using SWLS mean scores, and adding a broader set of sociodemographic covariates. For comparison, the model that adjusted the outcome for T1 SWLS only produced larger indirect effects, illustrating that inclusion of the immediately preceding T2 outcome yields a more conservative estimate without eliminating the two focal pathways.

**Table 14:** Sensitivity analyses.

Analysis	*N*	Direct effect	Indirect via BI	Indirect via PACES	Combined focal indirect
Primary model	426	−0.414	−0.150	−0.237	−0.387
Without MVPA and PA level	426	−0.438	−0.181	−0.312	−0.494
Excluding T1 SBQ outliers ±3 SD	424	−0.346	−0.148	−0.263	−0.410
Alternative outcome: SWLS mean	426	−0.083	−0.030	−0.047	−0.077
Expanded sociodemographic covariates	426	−0.417	−0.159	−0.239	−0.398
Original outcome adjustment (T1 SWLS only)	426	−0.410	−0.193	−0.288	−0.482

## 4 Discussion

### 4.1 Principal findings

The present study examined the longitudinal association between sedentary behavior and life satisfaction among college students, focusing on the parallel mediating roles of body image and physical activity enjoyment. The mediation model controlled both T2 and T1 life satisfaction, thereby providing stronger temporal adjustment of the outcome. Baseline sedentary behavior remained negatively associated with T3 life satisfaction, and both BI and PACES carried significant indirect effects. These findings support two complementary psychological pathways linking sedentary behavior with subsequent wellbeing while providing conservative estimates of their magnitude.

The wave-specific measurement models showed close fit, with parcel construction and factor loadings reported transparently. Longitudinal invariance was more nuanced: BI supported full metric and partial scalar invariance; PACES required partial metric invariance and did not support scalar invariance; and SWLS supported partial metric and partial scalar invariance. These results permit cautious interpretation of longitudinal structural associations but do not support unrestricted latent mean comparisons for all constructs. The fully reciprocal manifest-score CLPM additionally showed that several associations were bidirectional, while the full-forward robustness model indicated that the mediator-to-outcome pathways were more stable than the direct adjacent-wave SBQ → SWLS path.

### 4.2 Sedentary behavior and life satisfaction

Consistent with Hypothesis 1, T1 sedentary behavior remained negatively associated with T3 life satisfaction after adjustment for T2 and T1 SWLS and the behavioral/demographic covariates (β = −0.095, *p* = 0.013). However, the full-forward CLPM attenuated the adjacent T2 SBQ → T3 SWLS path to nonsignificance after T1 → T3 lag-2 effects were added. The direct longitudinal association should therefore be interpreted cautiously, whereas the indirect pathways through body image and physical activity enjoyment were more robust across model specifications.

College students often experience prolonged sitting due to academic learning, digital device use, online entertainment, and examination preparation. Systematic review evidence has shown that university students accumulate substantial daily sedentary time, with self-reported and device-based estimates indicating high levels of sitting across this population (1). Although these activities may appear routine, extended sedentary time may gradually weaken students' perceived vitality, reduce opportunities for social and physical engagement, and contribute to a less active lifestyle rhythm. Such changes may, in turn, reduce students' overall evaluation of their quality of life. The present results therefore extend previous work on student wellbeing by showing that sedentary behavior has prospective relevance for life satisfaction, even when baseline life satisfaction is considered.

It is also important to note that sedentary behavior should not be understood simply as the opposite of physical activity. A student may engage in some moderate-to-vigorous physical activity but still spend most of the day sitting. This distinction has been emphasized in sedentary behavior research, which suggests that sedentary behavior and physical inactivity represent related but conceptually different behavioral constructs (2, 3). In the present study, the association between sedentary behavior and later life satisfaction remained meaningful after physical activity level was controlled. This supports the theoretical distinction between insufficient physical activity and excessive sedentary behavior and suggests that reducing prolonged sitting may have unique value in university health promotion.

### 4.3 Mediating role of body image

Hypothesis 2 was supported. Body image significantly mediated the longitudinal association between sedentary behavior and life satisfaction even after the revised model controlled the immediately preceding T2 SWLS score. The specific indirect effect was *b* = −0.150 [95% CI (−0.247, −0.079)]. This persistence under stronger temporal adjustment suggests that body-related self-evaluation contributes explanatory information beyond prior life-satisfaction stability.

Body image is a multidimensional construct involving perceptions, attitudes, and emotional evaluations toward the body (7). During university years, students are often exposed to appearance-related social comparison, fitness ideals, and peer evaluation. Prior research has shown that body dissatisfaction, appearance concerns, and body appreciation differ across gender and age groups, indicating that body-related self-evaluation is an important psychological issue across developmental stages (29). In this context, prolonged sedentary behavior may contribute to negative body-related self-perceptions by reducing perceived physical competence, increasing concerns about body shape or weight, and weakening the sense of being physically active and capable. Once negative body image develops, it may influence broader self-evaluation and reduce satisfaction with life.

The present finding also highlights the psychological significance of the body in subjective wellbeing. Life satisfaction is not determined only by external academic or social conditions; it is also shaped by how individuals experience and evaluate themselves. A more positive body image may support self-acceptance, confidence, and emotional security, whereas negative body image may increase self-consciousness and dissatisfaction. Previous research among college students has shown that body image can mediate the relationship between physical exercise and subjective wellbeing (10). Therefore, body image appears to be an important self-evaluative pathway through which sedentary behavior is linked to college students' life satisfaction.

### 4.4 Mediating role of physical activity enjoyment

Hypothesis 3 was also supported. Physical activity enjoyment significantly mediated the association between sedentary behavior and later life satisfaction after adjustment for both T2 and T1 SWLS. The specific indirect effect was *b* = −0.237 [95% CI (−0.368, −0.141)], indicating that exercise-related affective experience remained an independent prospective pathway under the more stringent longitudinal specification.

Physical activity enjoyment reflects the extent to which individuals experience pleasure, interest, satisfaction, and intrinsic reward during movement. The Physical Activity Enjoyment Scale was developed to assess enjoyment derived from physical activity and has been widely used in exercise psychology research (11). From the perspective of self-determination theory, enjoyable physical activity is more likely to be intrinsically motivated and sustained over time. Self-determination theory emphasizes that autonomy, competence, and relatedness are central to intrinsic motivation and psychological wellbeing (12). In the exercise domain, systematic review evidence further suggests that autonomous motivation and intrinsic forms of regulation are positively associated with physical activity participation and maintenance (13).

The present results therefore suggest that exercise-related affective experience is a meaningful mechanism linking sedentary behavior to life satisfaction. Students with a highly sedentary lifestyle may feel less comfortable, less competent, or less motivated during physical activity, which may reduce enjoyment and make active behavior less likely to be maintained. Conversely, students who enjoy physical activity are more likely to associate movement with pleasure rather than obligation. Empirical evidence also indicates that enjoyment is associated with exercise habit, intention to continue exercising, and exercise frequency (14). For university health promotion, this finding suggests that interventions should focus not only on increasing exercise participation but also on improving the quality and enjoyment of students' movement experiences.

### 4.5 Parallel mediation: two distinct psychological pathways

Hypothesis 4 was supported. Body image and physical activity enjoyment operated as significant parallel mediators between sedentary behavior and life satisfaction. This finding provides a more nuanced explanation of how sedentary behavior may affect wellbeing. Rather than acting through a single pathway, sedentary behavior appears to be associated with life satisfaction through at least two distinct psychological mechanisms.

The body image pathway reflects a self-evaluative mechanism. It concerns how students perceive and judge their own bodies. In this pathway, prolonged sedentary behavior may be associated with less positive body-related self-evaluation, which subsequently reduces life satisfaction. In contrast, the physical activity enjoyment pathway reflects an affective-motivational mechanism. It concerns how students emotionally experience movement and exercise. This conceptual distinction is consistent with the view that body image involves self-perception and body-related attitudes, whereas exercise enjoyment reflects affective and motivational responses to physical activity (7, 11, 12).

The comparison of indirect effects showed that both mediating pathways were significant, while their difference was not statistically significant [difference = 0.088, 95% CI (−0.040, 0.237)]. Body image accounted for 38.6% and physical activity enjoyment for 61.4% of the combined focal indirect effect. Accordingly, the slightly larger PACES pathway should not be interpreted as statistically stronger; the two mechanisms are better viewed as complementary targets for health-promotion strategies.

### 4.6 Cross-lagged evidence and temporal direction

The fully reciprocal manifest-score CLPM provided a more transparent test of prospective ordering. All four constructs showed moderate-to-strong autoregressive stability, while cross-lagged paths were estimated in both directions for every construct pair. Sedentary behavior prospectively predicted lower BI across both adjacent intervals and lower PACES from T1 → T2. Conversely, BI also predicted lower subsequent sedentary behavior at both intervals, demonstrating that the longitudinal association was not exclusively one-directional. BI and PACES consistently predicted later SWLS, whereas several other reciprocal paths were small or nonsignificant.

These results support prospective between-person ordering but not within-person causal change. In particular, the full-forward model showed that the T2 BI → T3 SWLS and T2 PACES → T3 SWLS paths remained significant after all T1 → T3 lag-2 effects were added, whereas the adjacent T2 SBQ → T3 SWLS path was attenuated to nonsignificance. This robustness pattern strengthens confidence in the mediator-to-outcome associations while suggesting that the direct SBQ–SWLS relation is more sensitive to model specification.

The findings should not be interpreted as definitive evidence of causality. Traditional CLPMs do not separate stable between-person differences from within-person fluctuations and cannot eliminate unmeasured confounding (24). The full-forward model addresses one specification concern by incorporating lag-2 effects, but a random-intercept CLPM or another within-/between-person decomposition would be required for claims about within-person processes. Academic stress, sleep quality, social support, depressive symptoms, and digital-media dependence also remain plausible unmeasured confounders.

### 4.7 Measurement quality and methodological implications

The reliability analyses showed that body image, physical activity enjoyment, and life satisfaction had acceptable to excellent internal consistency across the three waves. The confirmatory factor analyses also supported the distinctiveness of the major psychological constructs, as the hypothesized multi-factor models fit the data substantially better than the one-factor models. These results indicate that body image, physical activity enjoyment, and life satisfaction should be treated as related but conceptually separable constructs.

The longitudinal measurement-invariance analyses now provide a more specific basis for interpretation. BI supported full metric invariance and partial scalar invariance. PACES required partial metric invariance after releasing two loading constraints, and scalar invariance was not supported; thus, PACES latent mean comparisons are not warranted. SWLS supported partial metric and partial scalar invariance after limited loading/intercept constraints were released. These results illustrate why invariance should be evaluated against multiple fit-change criteria and why the exact constraints must be reported rather than relying on global fit alone (21, 22, 30).

The common-method diagnostics should likewise be interpreted cautiously. A first unrotated factor below 40% and a poorly fitting one-factor CFA indicate that a single dominant factor does not adequately summarize the covariance among the measures, but neither procedure establishes the absence of common method bias (23). Temporal separation across waves and the distinction among measurement factors reduce some concerns, yet shared self-report method variance may still be present.

### 4.8 Practical implications

From a public health and health-promotion perspective, the findings support an integrated approach to student wellbeing in university settings. Sedentary behavior should be treated as a modifiable target distinct from insufficient physical activity, with feasible strategies such as regular movement breaks, active-learning practices, and prompts to interrupt prolonged study- and screen-related sitting. Because body image and physical activity enjoyment mediated the association with life satisfaction, sedentary-time reduction should be combined with non-stigmatizing body-image education and autonomy-supportive, enjoyable movement opportunities. Emphasizing body functionality rather than appearance and offering diverse, low-barrier physical activities may improve acceptability and sustained engagement. Overall, campus health promotion may benefit from addressing behavior, self-perception, and affective experience together rather than relying solely on exercise prescriptions (2, 12, 13, 31).

### 4.9 Limitations and future directions

Several limitations should be acknowledged. First, although the longitudinal design strengthens temporal ordering, the study remains observational and the mediation/CLPM coefficients should not be interpreted causally. The full-forward robustness model reduced the adjacent SBQ → SWLS coefficient, underscoring the sensitivity of direct cross-lagged effects to model specification. In addition, the manifest-score CLPM does not distinguish within-person fluctuations from stable between-person differences; future work should consider RI-CLPM or related models when the substantive question is explicitly within-person (24).

Second, sedentary behavior was assessed using self-report questionnaires. Self-reported sitting time may be affected by recall bias and social desirability. Future studies should combine self-report measures with device-based assessments, such as accelerometers or posture sensors, to obtain more accurate estimates of sedentary time and sitting patterns.

Third, the study focused on total sedentary time, but different types of sedentary behavior may have different psychological meanings. Academic sitting, passive screen use, social media use, gaming, and transportation-related sitting may not have equivalent effects on body image or life satisfaction. Previous research on university students has emphasized that sedentary behavior is shaped by multiple intrapersonal, interpersonal, environmental, and temporal factors (32). Future research should distinguish between mentally active, socially connected, and passive sedentary contexts.

Fourth, although body image and physical activity enjoyment were examined as parallel mediators, other mechanisms may also be involved. Sleep quality, perceived stress, depressive symptoms, loneliness, self-esteem, physical fitness, and social connectedness may further explain the association between sedentary behavior and wellbeing. Future studies could develop more comprehensive models integrating behavioral, emotional, cognitive, and social pathways.

Fifth, the sample was drawn from college students in one regional context, limiting generalizability to other provinces, countries, educational systems, or non-student young adults. The inferential analyses also relied on the 426 matched complete cases. Although no item-level data were missing within this analytic sample, the submitted database did not include the wave-specific records of participants who were lost before T3; therefore, FIML using all 526/476 available observations and retained-vs.-attrited baseline comparisons could not be reconstructed. Attrition bias cannot be ruled out and should be examined in future studies that retain all wave-specific records.

Finally, measurement invariance was only partial for some constructs. BI showed partial scalar invariance, SWLS showed partial metric and partial scalar invariance, and PACES did not achieve scalar invariance. These findings support cautious longitudinal structural interpretation but preclude unrestricted mean-level comparisons for all constructs. Future studies should replicate the parcel/item structure and test invariance prospectively in independent samples.

## 5 Conclusions

This study provides three-wave longitudinal evidence that sedentary behavior is prospectively associated with college students' life satisfaction and that body image and physical activity enjoyment represent two significant parallel explanatory pathways. Both indirect effects remained significant after controlling the immediately preceding T2 life-satisfaction score as well as baseline life satisfaction, supporting a more conservative longitudinal mediation interpretation. The fully reciprocal and full-forward panel analyses further indicate that the mediator-to-outcome pathways are more robust than the direct adjacent-wave sedentary-behavior effect.

From a practical perspective, the results highlight the need for university health promotion programs that move beyond simply encouraging exercise. Reducing prolonged sitting, fostering positive body image, and creating enjoyable physical activity experiences may jointly contribute to improved life satisfaction among college students. Future research using objective sedentary behavior measures, intervention designs, and more diverse samples will help clarify the causal mechanisms underlying these associations.

## Data Availability

The original contributions presented in the study are included in the article/Supplementary material, further inquiries can be directed to the corresponding author.
